# Cytocompatibility, Antibacterial, and Anti-Biofilm Efficacy of Grape Seed Extract and Quercetin Hydrogels Against a Mature Endodontic Biofilm Ex Vivo Model

**DOI:** 10.3390/jcm13216464

**Published:** 2024-10-28

**Authors:** Huda Mohammed Ahmed Aqabat, Mohamed Abouelseoud, Shereen N. Rafaat, Mohamed Shamel, Edgar Schäfer, Erick Miranda Souza, Shehabeldin Saber

**Affiliations:** 1 Department of Endodontics, Faculty of Dentistry, The British University in Egypt (BUE), El Sherouk City 11837, Egypt; h_akabat@hotmail.com (H.M.A.A.); mohammed.abouelseoud@bue.edu.eg (M.A.); 2 Department of Pharmacology, Faculty of Dentistry, The British University in Egypt (BUE), El Sherouk City 11837, Egypt; shereen.nader@bue.edu.eg; 3 Dental Science Research Group, Health Research Centre of Excellence, The British University in Egypt (BUE), El Sherouk City 11837, Egypt; mohamed.shamel@bue.edu.eg; 4 Department of Oral Biology, Faculty of Dentistry, The British University in Egypt (BUE), El Sherouk City 11837, Egypt; 5 Central Interdisciplinary Ambulance, School of Dentistry, University of Münster, 48149 Münster, Germany; 6 Department of Dentistry II, Federal University of Maranhão, São Luis 65080-805, Brazil; erickmsouza@uol.com.br

**Keywords:** cytocompatibility, grape seed extract, quercetin, hydrogels, calcium hydroxide, intracanal medication, apical periodontitis, endodontic biofilms, enterococcus faecalis

## Abstract

**Background/Objectives**: To assess the cytocompatibility, antibacterial and anti-biofilm efficacy of grape seed extract (GSE) and quercetin hydrogels versus calcium hydroxide (CH) as intracanal medications (ICMs) against an endodontic ex vivo biofilm model. **Methods**: Single-rooted teeth (*n* = 50) were prepared and sterilized before being infected with *E. faecalis* to develop a mature biofilm. They were divided into five equal groups according to the ICM used: G1: medicated with CH paste, G2: medicated with GSE hydrogel, G3: medicated with quercetin hydrogel, G4: positive control group that was infected and not medicated, and G5: negative control group that was neither infected nor medicated. After 1 week, the ICM was removed, and the root canals were cultured to assess the antibacterial efficacy by counting the colony-forming units and the anti-biofilm efficacy by the crystal violet assay. Dead/live bacterial viability was assessed by CFLSM examination, while the cytocompatibility was assessed using the MTT assay. **Results**: CH had the best antibacterial efficacy, followed by GSE and quercetin hydrogels (*p* < 0.001). Regarding the anti-biofilm efficacy, GSE was superior, followed by quercetin and CH (*p* < 0.001). CFLSM examination showed CH and GSE hydrogel to be highly effective in comparison to the positive control (*p* < 0.0001), with no statistical difference between them (*p* > 0.05). CH showed significantly higher cell viability percentages using a 500 μg/mL, while quercetin and GSE started to show cell viability > 70% at concentrations of 125 μg/mL and 62.5 μg/mL. **Conclusions**: CH fulfilled the ideal requirements of ICM as being both antibacterial and non-cytotoxic compared to the other materials tested.

## 1. Introduction

The principal objective of endodontic treatment is to avoid and treat “apical periodontitis” associated with unfavorable inflammatory destruction of the periapical tissues as a consequence of microbial infection [1]. Successful treatment outcomes rely on the thorough chemical cleaning and mechanical shaping of infected root canals [2], followed by their adequate sealing [3]. The disinfection phase is challenged by the intricate root canal anatomy. The presence of inaccessible regions within root canals offers shelter for the development, maturation, and establishment of bacterial biofilms [4], rendering shaping alone inadequate to clean canals [5]. Hence, the use intracanal medications (ICMs) is recommended to increase bacterial elimination [6].

The ICM frequently used is calcium hydroxide (CH) [7]. The mechanism of CH antimicrobial activity is attributed to its alkalinity, which inhibits microbial growth [8]. However, clinically, it might have an attenuated effect due to its inability to penetrate infected dentinal tubules [9], dentine buffering [10] or the presence of resistant strains linked to refractory apical periodontitis [11].

Alternative ICMs have been advocated, including aldehydes, halides, steroids, chlorhexidine, phenolics, and antibiotics [12]. Herbal products are recommended for use due to their compatibility with biological systems, along with their anti-microbial, anti-oxidant, and anti-inflammatory effects [13]. According to the World Health Organization, herbal medicine refers to preparations or materials derived from plants, which may include either processed or raw elements from various plants and possess medicinal qualities [14].

Grape seed extract (GSE) or Vitis vinifera is a bioavailable source of proanthocyanidins (PACs) that is recognized for its promising pharmacological effects [15]. As GSE improves the mechanical properties of dentin, it can be considered for use as an ICM [16].

Quercetin (3,3′,4′,5,7-pentahydroxyflavone) is a flavonol sourced from plants, and is noted for its anti-cancer, anti-inflammatory, anti-oxidant, and anti-allergic properties [17]. Furthermore, it has been shown to be a potent anti-microbial agent that is effective against a wide array of pathogenic bacteria [18].

Recent research aimed at creating bioactive substances that enhance the release of functional molecules into adjacent tissues [19]. This can be attained by integrating such materials into hydrogels, which provide tunable properties, targeted delivery, excellent biocompatibility, and high loading capacity [20].

From a clinical standpoint, the ICM used in endodontic treatment should be antibacterial, i.e., eliminating or significantly reducing the microbial load within root canals to create an environment favorable for healing [21], while maintaining cytocompatibility, as they can likely interact with the periradicular tissues, negatively affecting undifferentiated cells [22].

Limited knowledge is available on the anti-microbial efficacy and cytocompatibility of GSE and quercetin prepared as hydrogels for use as endodontic ICM. Thus, it was the aim of this study to evaluate and compare the cytocompatibility, antibacterial, and anti-biofilm efficacy of GSE and quercetin hydrogels versus the standard ICM CH against a mature endodontic ex vivo biofilm model. The null hypothesis tested was that there would be no differences between the tested materials.

## 2. Materials and Methods

### 2.1. Sample Size Calculation

A power analysis was conducted to ensure adequate power to statistically test the null hypothesis that there is no difference between tested groups. According to the results of Varshini et al. [23], with an effect size (f) of (0.649) and by adopting an alpha level and beta levels of 0.05, i.e., power = 95%, the predicted sample size (n) was a total of 50 samples to study the antibacterial and the anti-biofilm effects. Sample size calculation was performed using an ANOVA F-test in G*Power software, version 3.1.9.7 (Heinrich-Heine University, Düsseldorf, Germany).

### 2.2. Sample Preparation

**Figure 1:**
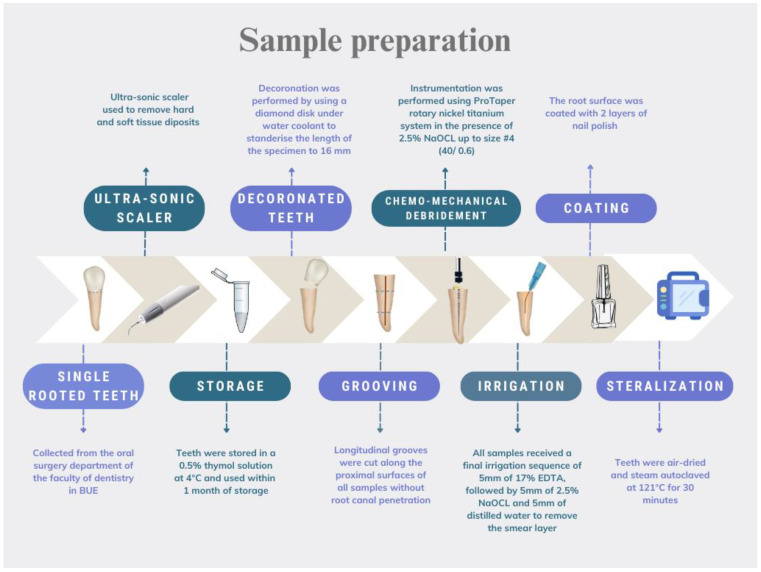
Flowchart illustrating the sample preparation.

Single-rooted teeth were collected from the Oral Surgery department at the British University in Egypt. For anatomic standardization, only mandibular premolars collected from young patients undergoing orthodontic treatment were include in this study. All teeth were single-canalled. They all had comparable overall length, as well as mesial–distal and buccal–lingual coronal measurements. An ultrasonic scaler (UDS-K LED, Woodpecker, China) operated at medium power cleaned all hard and soft tissue deposits. Teeth were stored in a 0.5% thymol solution at 4 °C and used within one month of storage. The teeth were de-coronated to a standardized length of 16 mm. Grooves were cut along the proximal surfaces of all the samples without root canal penetration. ProTaper (Dentsply Maillefer, Ballaigues, Switzerland) rotary nickel titanium instruments were used for canal preparation, up to size 40/0.6, in the presence of 2.5% NaOCl (JK dental vision, Cairo, Egypt). Finally, the smear layer was removed, and the teeth were dried and autoclaved after coating the root surface with nail polish. A flowchart showing the sample preparation procedures is presented in Figure 1.

### 2.3. Preparation of GSE and Quercetin Hydrogels

An amount of 150 mg of Carbopol (Lubrizol, Wickliffe, OH, USA) was soaked in 1.25 mL of distilled water for 30 min in a beaker to obtain a gel base. An amount of 250 mg of quercetin extract (Nawah, Cairo, Egypt) or 62.5 mg of grape seed extract (Puritan’s Pride, Long Island, NY, USA) was separately dissolved in 1 mL of ethanol (Merck, Darmstadt, Germany). The prepared extract solution was added to Carbopol gel base and stirred slowly. One drop of triethanolamine (Nerol, Cairo, Egypt) was added to maintain the pH and the volume was adjusted to 5 mL with distilled water. The final concentrations of Carbopol, GSE, and quercetin extracts were 3% *w*/*v*, 1.25% *w*/*v*, and 5% *w*/*v*, respectively.

Determination of minimum inhibitory concentration (MIC), which is the lowest concentration of the antimicrobial agent that inhibits visible microbial growth, was determined according to the Clinical and Laboratory Standards Institute (CLSI) guidelines [24] using the microtitre broth dilution method after overnight incubation with *E. faecalis* (ATCC29212) at 37 °C.

Determination of minimum bactericidal concentration (MBC) was considered to be the lowest concentration of the antimicrobial agent that killed 99.9% of the bacteria after overnight incubation at 37 °C [25].

Determination of minimum inhibitory biofilm concentration (MIBC), which is the lowest concentration of the antimicrobial agent to inhibit the initial formation of a biofilm, was assessed by the microtiter plate assay against sublethal concentrations (75, 50, and 25%) of previously determined MBC [26].

### 2.4. Classification of the Samples

The teeth were randomly divided into five groups (n = 10) according to the ICM, as follows: G1 (CH): medicated with aqueous CH paste; G2 (GSE): medicated with GSE hydrogel; G3 (quercetin): medicated with quercetin hydrogel; G4: positive control group that was infected and not medicated; and G5: negative control group that was neither infected nor medicated but cultured at the end of the experiment to check for the sterility of the procedures.

### 2.5. Microbiologic Assessments

For the CH, GSE, and quercetin groups, as well as for the positive control group, root canals were incubated with a 24 h pure culture suspension of *E. faecalis* (ATCC29212) at 37 °C for 1 week to develop a mature biofilm. The infected culture was cultivated in Brain Heart Infusion broth (BHI; Difco Laboratories, Detroit, MI, USA) adjusted to No. 1 MacFarland turbidity standard. Every 72 h, this procedure was repeated by using a 24 h pure culture that was prepared and modified to the No. 1 MacFarland turbidity standard. The negative control samples were immersed in sterile BHI broth that was replenished every 72 h with sterile saline. At the end of the incubation period, the root canals of the experimental groups were medicated with either an aqueous CH paste (Meta Biomed, Seoul, Korea), GSE, or quercetin hydrogels and sealed coronally with sticky wax, while the root canals of the positive control group were sealed without being medicated. After 1 week, the ICM was removed by copious saline irrigation (20 mL/canal), and the root canals were cultured to evaluate the antibacterial efficacy of the different ICMs by counting the colony-forming units (CFUs), as described previously [27], and also to assess the ability of the bacterial strain to develop a biofilm by the crystal violet assay, as described previously [28].

### 2.6. CFLSM Examination

Dead/live bacterial viability was assessed by CFLSM examination [29]. The root segments were split into 2 pieces using a hammer and chisel, and the most preserved half of each sample was used for evaluations. The dual-dye method was used, in which 10 microliters of Acridine Orange (AO) stain (100 µg/mL) (Sigma-Aldrich, St. Louis, MO, USA) and 20 microliters of Propidium Iodide (PI) stain (100 µg/mL) (Sigma-Aldrich) were combined using a Thermo Scientific™ LP Vortex Mixer (Fisher Scientific, Waltham, MA, USA) for 30 s. After mixing the dye solution, the selected sections were stained with the mixture and kept in a dark room at 25 °C for 15 min. Finally, the stained sections were washed with sterile saline three times and placed on a microscope glass slide. CLSM images were acquired from a region of interest, which was the apical 2 mm of the root canal, using Leica Application Suite Leica DMi 8 (Leica stellaris 5 true confocal microscope upgrade set for Leica DMi 8 fluorescence microscope) includes the Stellaris 5 confocal package, which contains the Stellaris 5 System, 2 Power HyD S laser lines, 3 VIS laser lines (488 nm, 562 nm, 638 nm), a 405 nm laser, a motorized stage, CS2PLAN APO objectives (10× and 63×), LAS X STELLARIS control software version 3.3 with workstation and monitor, Leica Microsystems, (Wetzlar, Germany) at ×40 magnification and a resolution of 1024 × 1024 pixels. For analysis, the images were imported into Fiji (ImageJ, US National Institutes of Health, Bethesda, MD, USA). Each confocal stack was processed by separating channels for live and dead stains, followed by applying thresholding techniques to convert grayscale images into binary representations for accurate particle analysis. The ‘Analyze Particles’ function in Fiji was employed to quantify the number of live and dead bacterial particles, ensuring the size and shape criteria were set to exclude non-specific signals. The percentages of live bacteria in the stacked images were calculated using the following formula: percentage of live bacteria (%) = (number of live bacteria particles/total number of bacteria particles (live + dead)) × 100 [30].

### 2.7. Cytotoxicity Test

Periodontal ligament (PDL) tissues were removed from the surface of the roots of the extracted teeth and cultured by the outgrowth method, as described previously [31]. The fourth passage cells were characterized as mesenchymal stem cells by flow cytometry [32] and confirmed for multilineage differentiation [22]. The cytotoxicity of the tested ICM to the human periodontal ligament stem cells (hPDLSCs) was assessed using the 3-(4,5-dimethylthiazol-2-yl)-2,5-diphenyltetrazolium bromide (MTT) assay, as described previously [33]. Briefly, 1 × 104 hPDLSCs were added to 96-well plates with 180 μL of Dulbecco’s Modified Eagle Medium (DMEM) for 24 h. Then, serial dilutions of the materials were added, including 1000 μg/mL, 500 μg/mL, 250 μg/mL, 125 μg/mL, 62.5 μg/mL, 31.25 μg/mL, 15.6 μg/mL, 7.8 μg/mL, and 3.9 μg/mL, respectively. The cells were incubated at 37 °C in a 5% CO_2_ for 1, 3, or 7 days. At the indicated time periods, the medium was removed, and the cells were incubated with 1 mg/mL of MTT for 4 h. Then, 0.2 mL of dimethyl sulfoxide (DMSO) was added to each well to solubilize the formazan crystals obtained as a result of MTT reduction by the viable cells. The optical density (OD) value was measured by spectrophotometer at 570 nm [34]. The data obtained by each group were normalized based on cells + medium, and cell viability was calculated using the following formula: (OD values of experimental wells/OD values of control wells × 100) [35]. All samples were analyzed in triplicate, and the experiment was repeated three times.

Cytotoxicity classification: Grade 0 (non-cytotoxic): cell viability ≥ 70%, Grade 1 (slightly cytotoxic): cell viability 50–69%, Grade 2 (moderately cytotoxic): cell viability 30–49%, Grade 3 (severely cytotoxic): cell viability < 30%.

Acceptance criteria: for an ICM to be considered cytocompatible, it should be classified as Grade 0 (non-cytotoxic) or Grade 1 (slightly cytotoxic) according to the cytotoxicity scale. A Grade 2 (moderately cytotoxic) or Grade 3 (severely cytotoxic) classification would indicate that the ICM does not meet the biocompatibility requirements.

### 2.8. Statistical Analysis

Numerical data were represented as mean and standard deviation (SD) values. Normality and variance homogeneity assumptions were verified by viewing the data distribution and using Shapiro–Wilk’s and Levene’s tests, respectively. All data were normally distributed (Shapiro–Wilk, *p* > 0.05). The assumption of homogeneity was violated for the bacterial count data, so they were analyzed using Welch-one ANOVA followed by the Games–Howell post hoc test. However, for other data, the assumption was valid. Cytocompatibility data were analyzed using a three-way ANOVA test. The comparisons of simple effects were made using the error term of the three-way model with *p*-value adjustment using the False Discovery Rate (FDR) method. Other data were analyzed using one-way ANOVA followed by Tukey’s post hoc test. The significance level was set at *p* < 0.05 within all tests. Statistical analysis was performed with R statistical analysis software version 4.4.1 for Windows (R Core Team (2024). R: A language and environment for statistical computing. R Foundation for Statistical Computing, Vienna, Austria. (URL: https://www.R-project.org/ accessed on 1 March 2022).

## 3. Results

### 3.1. MIC and MBC

The MIC of CH paste, GSE, and quercetin hydrogels against *E. faecalis* (ATCC 29212) were 41.667 µg/mL, 62.5 µg/mL, and 250 µg/mL, respectively. The MBC of CH paste, GSE, and quercetin hydrogels against *E. faecalis* (ATCC 29212) were 125 µg/mL, 500 µg/mL, and 125 µg/mL, respectively.

### 3.2. MBIC

The MBIC of sublethal concentrations of CH paste, GSE, and quercetin hydrogels against *E. faecalis* (ATCC 29212) are presented in Table 1.

### 3.3. Antibacterial and Anti-Biofilm Efficacy

Intergroup comparisons, mean and standard deviation (SD) values for log bacterial counts (CFU/mL), and the anti-biofilm activity (%) are presented in Table 2.

The lowest bacterial counts were achieved with CH paste, followed by GSE and quercetin hydrogels, respectively (*p* < 0.001). Regarding the anti-biofilm activity, GSE was superior, followed by quercetin and CH, respectively (*p* < 0.001).

### 3.4. CFLSM

**Figure 2:**
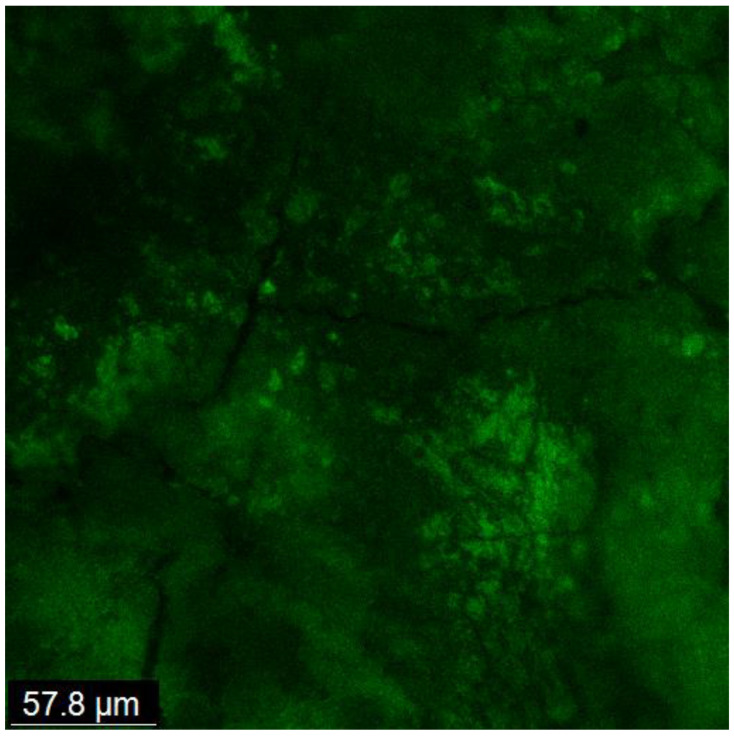
CFLSM examination showing the development and maturation of a thick biofilm matrix covering the entire dentin surface, with the absence of any visible dentinal tubules.

**Figure 3:**
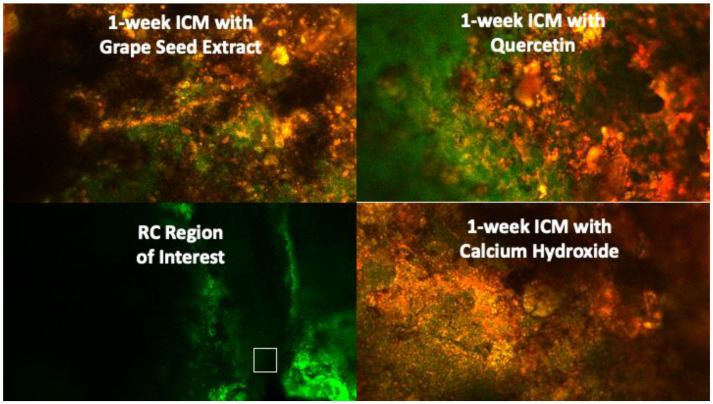
CFLSM representative images for the experimental groups at the region of interest (apical 2 mm of the root canal) after 1 week of intracanal application.

**Figure 4:**
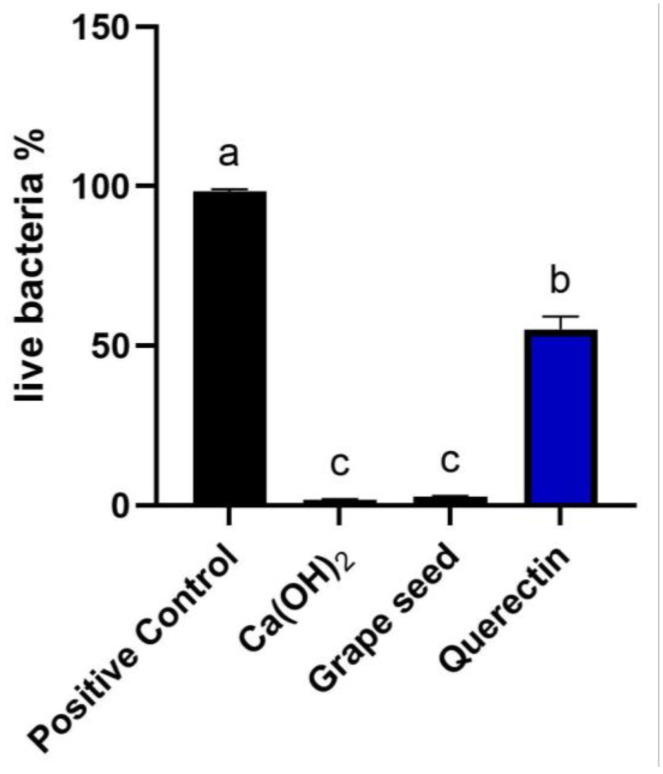
Comparative analysis of mean percentage of live bacterial cells for all groups. Different letters denote significant differences between groups (*p* < 0.05).

Biofilm development and maturation after 1 week were confirmed by CFLSM examination, as presented in Figure 2. Representative CFLSM images for the experimental groups are presented in Figure 3. Statistical analysis of the results, presented in Figure 4, showed a significant difference in the mean percentages of live bacteria among all groups (*p* = 0.0001). The post hoc analysis using Tukey’s test revealed that ICM with CH and GSE hydrogel were highly effective, with almost complete elimination of live bacteria. Both groups showed a significant decrease (*p* < 0.0001) in the percentage of live bacteria compared to the positive control, with no statistical difference between them (*p* > 0.05). Treatment with quercetin hydrogel showed intermediate effectiveness, but significantly less than CH and GSE treatments (*p* = 0.0001).

### 3.5. Cytotoxicity Assay

**Figure 5:**
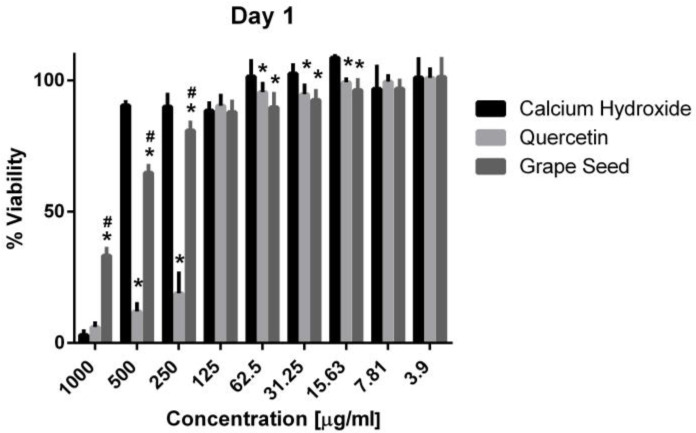
Mean, standard deviation, and comparative statistics of cell viability percentage of HPDLSCs after exposure to CH, GSE and Quercetin hydrogels at day 1. * indicates significant difference with CH, ^#^ indicates significant difference with Quercetin (*p* < 0.05).

**Figure 6:**
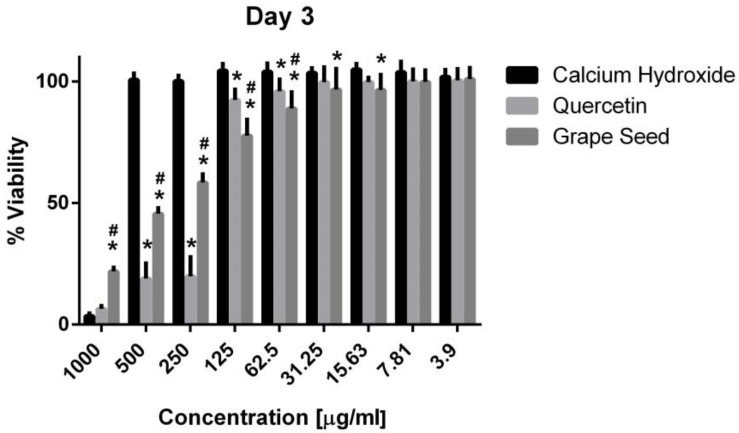
Mean, standard deviation, and comparative statistics of cell viability percentage of HPDLSCs after exposure to CH, GSE and Quercetin hydrogels at day 3. * indicates significant differences with CH, ^#^ indicates significant difference with Quercetin (*p* < 0.05).

**Figure 7:**
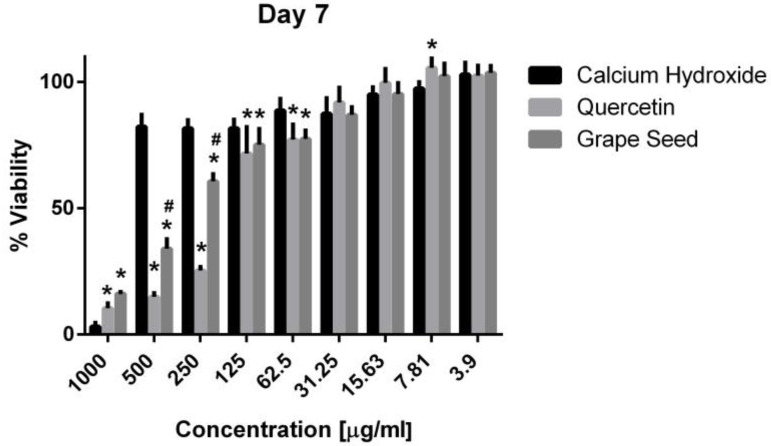
Mean, standard deviation, and comparative statistics of cell viability percentage of HPDLSCs after exposure to CH, GSE and Quercetin hydrogels at day 7. * indicates significant differences with CH, ^#^ indicates significant difference with Quercetin (*p* < 0.05).

Three-way ANOVA indicate that the variables medicaments, concentrations and time frames of observations significantly influence the cell viability (*p* = 0.001, for all variables). The interaction among the three variables was highly significant (*p* = 0.001), indicating that depending on the concentration and time frame observed, the medicaments present varying cell viability. The three materials showed cytotoxic effects on the hPDLSCs (viability < 70%) using a 1000 μg/mL concentration at the three time frames (*p* = 0.001). CH was associated with significantly higher cell viability percentages using a 500 μg/mL concentration compared to GSE and quercetin at the three time intervals (*p* = 0.001) (Figure 5, Figure 6 and Figure 7), with cell viability mean values 90.5 ± 1.28, 100.7 ± 2.5, and 82.3 ± 4.6 on days 1, 3 and 7, respectively. Quercetin and GSE started to show cell viability > 70% at concentrations of 125 μg/mL and 62.5 μg/mL, depending on the time frame observed. However, no significant difference was found between both materials using the same respective concentrations on day 1 and day 7 (*p* > 0.05). The three materials showed comparable cell viability (>85%) on days 1, 3 and 7 after being diluted to 31.25 μg/mL, 15.63 μg/mL, 7.81 μg/mL, and 3.9 μg/mL.

## 4. Discussion

According to Estrela et al. [36], endorsement of biofilm models used to assess the antibacterial effectiveness of endodontic materials require three key factors; human dentin as substrate, a common endodontic pathogen, and an adequate incubation period to allow for biofilm growth and maturation. Our model fulfilled all these factors. *E. faecalis*, an extensively evaluated endodontic pathogen, was the biological marker. Lastly, the incubation period was enough to allow for bacterial biofilm’s growth and maturity that was confirmed by CFLSM examination of the positive control group after 1 week. *E. faecalis* has been suggested as being commonly associated with post-treatment apical periodontitis [37]. However, recent reports using more sensitive culturing methodologies reported its existence as being less than expected [28].

Regarding the ICM selected for use in this study, CH was selected as the sole standard for comparison with the novel herbal agents. This is because CH is the most commonly used ICM worldwide, being one of the oldest, safest, and best investigated endodontic material.

The choice to use GSE and Quercetin as hydrogels was made to explore if this can improve their efficacy. Hydrogels have a three-dimensional network that can absorb water and swell. As water penetrates the hydrogel, it creates a concentration gradient that allows the active substances to move out of the gel into the surrounding environment [20]. This can allow better dentinal tubule penetration, especially with their relatively small molecular size. Another effect comes from the polyphenolic compounds present in GSE and Quercetin that can interact with the organic matrix of dentin, enhancing adhesion and retention and providing a sustained effect of inhibiting and disrupting microbial biofilms [38].

This study employed the culture method as a primary investigation method. This was augmented by the CRV assay to quantitatively assess the ability of the biological indicator to develop a biofilm. Moreover, CFLSM was used to assess the percentage of viable/dead bacteria after ICM application. The key principle of CFLSM is its ability to differentiate between dead and live bacteria through the permeability of the bacterial cell membrane. Live, viable bacteria have an intact cell membrane that selectively allows the passage of certain molecules in and out of the cell. In contrast, dead or compromised bacterial cells have a disrupted or permeable cell membrane.

The obtained results evidenced by the microbiologic and CFLSM assays showed that regarding the antibacterial efficacy, CH was the most efficient ICM, followed by GSE and Quercetin hydrogels, respectively. The superior performance of CH over GSE and Quercetin hydrogels can be attributed to its mechanism of drug release and pH. CH is highly water soluble, thus dissociates in aqueous media resulting in a rapid and burst-like release of hydroxyl ions, which create a highly alkaline environment. This extreme pH, typically around 12–13, is responsible for the potent antibacterial activity of CH, as it disrupts bacterial cell membranes and inhibits essential enzymatic activities. Conversely, the pH of GSE and Quercetin hydrogels is not alkaline, typically in the range of 5–7, making them more physiologically compatible and less damaging to the surrounding tissues. Moreover, the release of the active polyphenolic compounds in GSE and Quercetin from the hydrogel matrix is mediated by the swelling and gradual erosion of the hydrogel network, which might retard their interaction with the target bacteria, compared to CH [38].

The present results agree with those of Cecchin et al. [39], Souza et al. [40], and Furiga et al. [41] who reported that GSE-based dressings were effective in reducing intracanal microbial loads, especially Gram-positive bacteria such as *E. faecalis*.

Regarding the anti-biofilm efficacy, GSE showed the best inhibitory effect, followed by quercetin and CH, respectively. This can be attributed to their hydrogel networks which, in addition to providing a sustained prolonged release of active polyphenolic compounds [42,43], can physically entrap and immobilize bacterial cells [44] and create a local environment unfavorable for their growth [45]. The anti-biofilm effect of quercetin has been reported earlier by Liu et al. [46] and Qauyyum et al. [47] and explained on the basis of distressing bacterial physiology by disruption of the protein translation-elongation and protein folding pathways, hence inhibit bacterial translation from the planktonic state to the biofilm state.

From a clinical perspective, the ICM used in endodontic treatment should exhibit a potent antibacterial action without distressing the differentiated and undifferentiated cells in the surrounding microenvironment. The cell viability of hPDLSCs, in particular, was assessed, as they are likely to come into contact with extruded ICM in case of perforated root canals, open apices, or forceful application.

The cell viability assay evaluated the cytocompatibility of different concentration/dilutions of the tested ICM. This was performed to identify the drug concentration that will be both effective and cytocompatible. The present results showed that CH was non-cytotoxic to the HPDLSCs at all observation times, up to four times (4×) its MBC. The MBC of GSE hydrogel was non-cytotoxic after 1 day and slightly cytotoxic, with cell viability of 66% and 68%, after 3 and 7 days, respectively. This time-dependent cytotoxic effect of GSE can be attributed to the generation of reactive oxygen species (ROS) [48], inhibition of the cell renewal capacity [49], and activation of apoptotic signaling pathways [50].

Finally, the MBC of quercetin was severely cytotoxic, with cell viability of 15%, 22%, and 15% after 1, 3, and 7 days, respectively. This effect can be attributed to the induction of oxidative stress in the stem cells, which triggers apoptosis [51,52], and the disruption of mitochondrial activity [53]. In addition, it has been reported to alter the epigenetic landscape of stem cells, which can influence gene expression and stem cell differentiation [54], and cause cell cycle arrest [55].

Translating the present cytocompatibility results to clinical practice suggests that calcium hydroxide and quercetin are safe for clinical use in infected root canals at concentrations equivalent to 500 μg/mL and 62.5 μg/mL, respectively, while GSE demonstrated concentration-dependent and time-dependent stem cell cytotoxicity, rendering it a suboptimal choice as an ICM. The impact of quercetin use on dentin requires further investigations; therefore, CH remains the preferred ICM for infected root canals.

Regarding the limitations of this study, using a single bacterial strain in an ex vivo model may not accurately reflect the complex oral microbiota and does not take into consideration other bacteria associated with endodontic infections. This should be considered in future studies. Moreover, it is important to acknowledge that the efficacy of ICM used during endodontic treatment can differ in laboratory settings compared to clinical conditions. In vitro, the environment is highly controlled, with specific pH, temperature, and nutrient conditions. Conversely, the in vivo environment is more complex and is influenced by several factors, such as complex microbial interactions. The host immune response, the dentin’s buffering capacity due to the presence of phosphates, carbonates, and calcium ions, the availability of nutrients, and the presence of synergistic microbial interactions [56], all contribute to the complexity of the clinical environment. Therefore, the selection of optimal concentrations for the clinical application of ICM is important to ensure that their biological actions are not neutralized or inhibited, and, at the same time, the material remains cytocompatible. Moreover, extending the ICM application for more than 1 week would provide insights into assessing the long-term efficacy of the tested antimicrobial agents, as well as the long-term effects on the dentin substrate and hydrogels’ stability. Furthermore, in order to allow a reliable transformation of the results obtained from this laboratory study, controlled clinical trials are warranted.

## 5. Conclusions

Taking the limitations of this study into account, it can be concluded that CH and quercetin used in the treatment model to reduce microbial load in infected root canals fulfilled the ideal requirements of ICM as being both potent antibacterial and, at the same time, non-cytotoxic compared to GSE. Future investigations should consider assessing the long-term efficacy of the ICM against a multi-species mature biofilm.

## Figures and Tables

**Table 1:** In vitro biofilm inhibition activity of the tested samples against Enterococcus faecalis ATCC 29212 (mean inhibition (%) ± SD).

Concentration	Biofilm Inhibition (%) (Mean ± SD)			
	CaOH	GSE	Quercetin	*p*-Value
25% of MBC	75.88 ± 0.53 ^C^	89.88 ± 0.12 ^A^	86.14 ± 0.69 ^B^	<0.001 *
50% of MBC	89.07 ± 0.55 ^C^	96.46 ± 0.19 ^A^	92.98 ± 0.25 ^B^	<0.001 *
75% of MBC	95.77 ± 0.50 ^B^	98.34 ± 0.16 ^A^	95.70 ± 0.17 ^B^	<0.001 *

Values with different superscript letters within the same horizontal row are significantly different; * significant (*p* < 0.05).

**Table 2:** Intergroup comparisons, mean and standard deviation (SD) for log bacterial count (CFU/mL) and anti-biofilm activity (%) for all groups.

	CaOH	GSE	Quercetin	Positive Control	*p*-Value
Log bacterial count (CFU/mL)	5.25 ± 0.56 ^D^	6.47 ± 0.28 ^C^	7.86 ± 0.46 ^B^	12.44 ± 0.14 ^A^	<0.001 *
Anti-biofilm activity (%)	91.40 ± 0.15 ^C^	95.71 ± 0.22 ^A^	92.55 ± 0.22 ^B^	0.00 ± 0.00 ^D^	<0.001 *

Values with different superscript letters within the same horizontal row are significantly different; * significant (*p* < 0.05).

## Data Availability

Available upon reasonable request from the corresponding author.
